# Preparation of Chitosan—Graphene Oxide Composite Aerogel by Hydrothermal Method and Its Adsorption Property of Methyl Orange

**DOI:** 10.3390/polym12092169

**Published:** 2020-09-22

**Authors:** Wei Zhu, Xueliang Jiang, Fangjun Liu, Feng You, Chu Yao

**Affiliations:** 1 School of Materials Science and Engineering, Wuhan Institute of Technology, Wuhan 430205, China; witzhuwei@163.com (W.Z.); 15387182689@wit.edu.cn (F.L.); youfeng.mse@wit.edu.cn (F.Y.); chuyao@wit.edu.cn (C.Y.); 2 The College of Post and Telecommunication of WIT, Wuhan 430073, China; 3 Hubei Key Laboratory of Plasma Chemistry and Advanced Materials, Wuhan 430205, China; 4 Key Laboratory of Green Preparation and Application for Functional Materials, Ministry of Education, Hubei University, Wuhan 430062, China

**Keywords:** graphene oxide, chitosan, aerogel, hydrothermal method, adsorption

## Abstract

Graphene based aerogel has become one of the most likely functional adsorption materials that is applicable to purify various contaminated water sources, such as dye wastewater, because of its high porosity, structural stability, large specific surface area, and high adsorption capacity. In this study, chitosan and graphene oxide were first selected as the matrix to prepare the composite hydrogel through the hydrothermal method, which was further frozen and dried to obtain the target aerogel. The microscopic structures and adsorption capacity of the composite aerogel were then characterized by Fourier transform infrared spectroscopy (FT-IR), scanning electron microscopy (SEM), X-ray powder diffraction (XRD) and N_2_ (nitrogen) physical adsorption and desorption tests. The results show that the specific surface area of the composite aerogel was reached at 297.431 m^2^/g, which is higher than that of graphene oxide aerogel and chitosan aerogel. The aperture was reduced to about 3 nm. The adsorption rate of the composite aerogel for the methyl orange solution was as high as 97.2% at pH = 1, and the adsorption capacity was 48.6 mg/g. The adsorption process of the composite aerogel satisfies the Langmuir equation and can be described by the second-order adsorption kinetics. In addition, it is worth noting that this composite aerogel can provide a striking adsorption characteristic on methyl orange due to the combining effects from massive amino groups on chitosan and the structural conjugation of graphene oxide.

## 1. Introduction

With the improvement to the aesthetic requirements of products, dyes have been widely used in many fields. Tremendous dye wastewater will be continuously produced and released to the surrounding areas during the period of industrial production, which results in hazardous environmental pollution [1,2,3]. Therefore, it is very important to effectively remove dyes from polluted water sources.

Adsorption is one of the most promising and reliable techniques for removing pollutants from wastewater [4]. Graphene oxide aerogel is a three-dimensional network structure material with graphene as the cross-linked body and gas as the dispersion medium. Graphene oxide aerogel not only retains the nano-characteristics of graphene, but also solves the dispersion difficulties of graphene. It is also a new material that can be used as an adsorbent. Chitosan and chitosan-based materials are considered to be good adsorbents due to their unique high reactivity, chemical stability and high affinity [5,6]. By combining graphene with chitosan, the advantages of specific surface area and porosity of graphene materials can be fully utilized while retaining the adsorption capacity of chitosan itself. The interlayer of chitosan can reduce the overlaps between graphene sheets, inhibit agglomeration, increase the specific surface area of graphene and improve adsorption capacity [7].

In recent years, the relevant research into chitosan/graphene composites has been reported successively. For example, Liao et al. [8] prepared 3D porous graphene oxide/polydopamine/chitosan (GO/PDA/CS) aerogels for crosslinking PDA with CS and assembled it. The active site was conducive to the removal of U (VI), and the maximum adsorption capacity was up to 415.9 mg/g at 298 K. Omid Ghasemi et al. [9] used graphene oxide (GO), chitosan and glutaraldehyde as raw materials to prepare the composite aerogel with a maximum adsorption capacity of 48.22 g/g for oil products in seawater. Liu et al. [10] prepared the chitosan/graphene oxide(CS/GO) composite material by adding a small amount of GO as a matrix and crosslinking it with glutaraldehyde. The maximum adsorption capacities were 1076.649 mg/g for Au(III) and 216.920 mg/g for Pd(II), respectively. Sabzevari [11] cross-linked graphene oxide (GO) with chitosan and prepared a composite material (GO-LCT) for the adsorption of methylene blue (402.6 mg/g). Wang et al. [12] prepared porous chitosan aerogels doped with graphene oxide (CSGO aerogels) by crosslinking and freeze drying and used them as highly effective adsorbents for two azo dyes, methyl orange (MO) and amido black 10B (AB10B). However, the preparation methods in the most reported literatures require the addition of cross-linking agents, such as epicloropropane, formaldehyde or glutaraldehyde, which are highly toxic and the experimental methods are relatively complex. Therefore, it is also of great importance to develop a simple way to synthesize the target composite aerogels and to reveal and analyze their absorption mechanisms.

The preparation method adopted in this paper is the hydrothermal method, which is simple and does not require the addition of a cross-linking agent. The production process is green, environmentally friendly, and has a low production cost. The structure and adsorption properties of chitosan and graphene oxide (CS/GO) composite aerogels prepared by three methods (hydrothermal, chemical crosslinking and sol-gel) were compared. The microstructure, specific surface area and porosity of the CS/GO aerogels were systematically investigated. To this end, the adsorption mechanism, the adsorption model, and the desorption and recycling of the CS/GO aerogel will analyzed and developed, respectively.

## 2. Experimental

### 2.1. Raw Materials

Natural graphite powder (97%), potassium permanganate (99.9%), concentrated sulfuric acid (98%), hydrogen peroxide solution (5%), deacetylation chitosan, sodium ascorbate, glutaraldehyde, methanol, phosphoric acid, hydrohdloric acid, acetic acid, sodium hydroxide, and methyl orange (MO) were purchased from Sinopharm Chemical Regent Co. Ltd., Shanghai, China.

### 2.2. Preparation of Chitosan (CS) Aerogel by Sol-Gel Method

At first, the homogeneous chitosan solution was obtained by adding 3 g chitosan into 100 mL dilute hydrochloric acid (2.5 wt%). Subsequently, a certain amount of chitosan solution was slowly dropped into 1 mol/L NaOH solution (at a controlled rate of 60 drops/min) and then reacted for 120 min. The reaction product was filtered, washed with distilled water to pH = 7, and freeze-dried at −50 °C (48 h) to obtain the target CS aerogel.

### 2.3. Preparation of Graphene Oxide (GO) Aerogel by Hydrothermal Method

**Figure 1:**
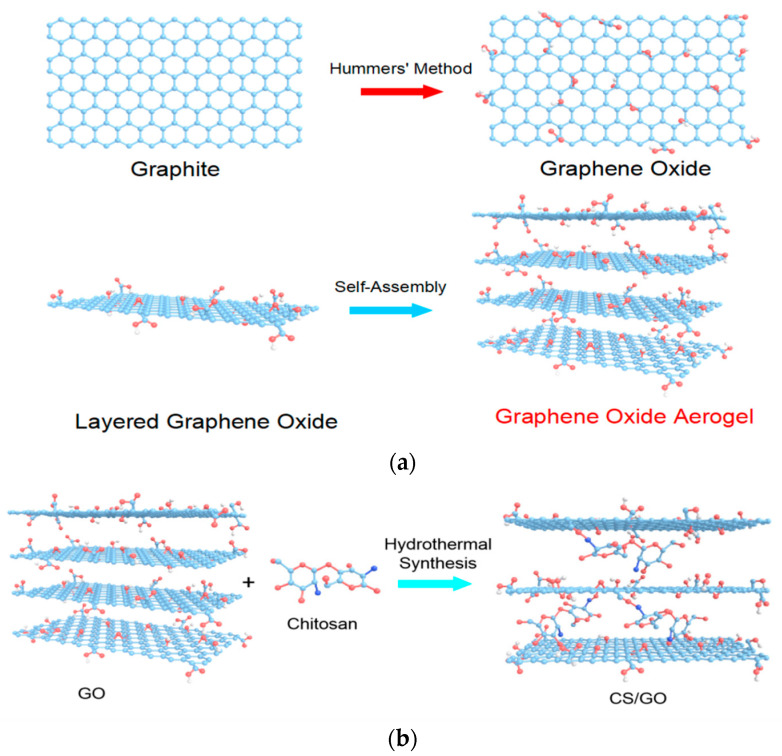
Schematic illustration of the synthesis mechanism: (**a**) GO aerogel, (**b**) CS/GO aerogel.

GO was prepared by the improved Hummers method and dispersed through an ultrasound in deionized water. 50 mL GO dispersion (4 mg/mL) was mixed with sodium ascorbate (the mass ratio is 1:1) and the mixture was then reacted in a hydrothermal kettle for 8 h at 120 °C. After this, the resulting products were freeze-dried at −50 °C (48 h) to obtain the GO aerogel. The schematic mechanism for the synthesis of the GO aerogel is shown in Figure 1a.

### 2.4. Preparation of Chitosan/Graphene Oxide (CS/GO) Composite Aerogel by Hydrothermal Method

A certain amount of chitosan was added to 100 mL acetic acid (2.5 wt%) and was put in an ultrasound for 2 h to completely dissolve the chitosan, forming a uniform light yellow transparent viscous liquid. Chitosan solutions of different qualities were added to the GO dispersion solution (4 mg/mL) so that the mass ratio of GO: CS was 3:1, 5:1, 10:1, 15:1, and 20:1. The mixture was added into the hydrothermal reaction kettle and reacted at 120 °C for 12 h to obtain the CS/GO hydrogel. After the CS/GO hydrogel was freeze-dried at −50 °C (48 h), the CS/GO aerogel was obtained. Figure 1b illustrates the synthesis mechanism of the CS/GO aerogel.

### 2.5. Preparation of CS/GO Aerogel by Chemical Crosslinking

Firstly, the pH value of GO dispersion was adjusted to 10 with the NaOH solution (1 mol/L), and then the GO dispersion was mixed with the chitosan solution (2.5 wt%). After 30 min of ultrasound, an appropriate amount of acetic acid was added to the mixture to adjust the pH to about seven. CS/GO hydrogel was obtained by adding glutaraldehyde solution and heating the mixture in a water bath at 95 °C for 24 h. Finally, the CS/GO aerogel was obtained by freeze-drying at −50 °C for 48 h.

### 2.6. Preparation of CS/GO Aerogel by Sol-Gel Method

0.0255 g GO was added into the 20 mL acetic acid solution (1%, *v*/*v*) and ultrasonic was used at room temperature for 30 min to form a uniform suspension. Then, 0.5 g chitosan powder was added, and the chitosan was completely dissolved by ultrasound for 1 h. After standing for 12 h, a syringe (pinhole for 0.7 mm) was used to drop 200 mL of the NaOH solution (3% *w*/*v*) into the suspension drop-by-drop (control speed 60 drops/min) to form a beadlike liquid. After standing for 24 h, the beads gradually solidified, then washed to neutral with deionized water, filtered and poured into a 250 mL flask. Then, 30 mL of methanol and 1.5 mL glutaraldehyde solution (50%, *v*/*v*) was added and stirred at room temperature for 5 h. After filtering the formed beads, they were washed with ethanol and deionized water three times, respectively, and freeze-dried at −50 °C for 48 h to obtain the grayish brown CS/GO composite aerogel.

### 2.7. Material Characterizations

Fourier transform infrared (FTIR) spectra were recorded on the Thermo Electron Nicolet 6700 spectrometer (Thermo Electron Corporation, Waltham, MA, USA) with the potassium bromide (KBr) pellet technique. The morphology and structure of the products were studied by a scanning electron microscope (SEM, JSM-5510LV, JEOL, Tokyo, Japan). The phase composition of the samples was determined using powder X-ray diffraction (XRD, Bruker D8-Advance, Cu Ka radiation, λ = 0.15418 nm, Bruker, Karlsruhe, Germany) at 40 kV and 20 mA. The ultraviolet-visible spectrometer (PerkinElmer, lambda35 Perkin Elmer, Waltham, MA, USA) was used to record the ultraviolet visible-light diffused reflection spectrums (UV-DRS) of the samples, and BaSO_4_ was used as the referential sample. A full automatic specific surface area and aperture distribution analyzer (Autosorb IQ, Quantachrome, Boynton Beach, FL, USA) obtained the adsorption and desorption isotherm curve and the corresponding pore size distribution of the sample at the nitrogen (N_2_) temperature, calculated the specific surface area by baxrett-emmett-teller (BET) method, and calculated the pore size distribution by the berret-joyner-halenda (BJH) model.

### 2.8. Adsorption Characterization

The adsorption activity of the samples was evaluated by measuring the adsorption rate of methyl orange (MO) solution. A certain concentration of methyl orange solution was prepared. Then, 50 mL of the solution, with initial concentrations, were shaken for 5 h at room temperature with 20 mg samples. Further, the pH was adjusted in the range of one to nine using HCl and NaOH solutions. After the specified time, the solid and liquid were separated immediately. The concentration (*C*_t_) of the centrifuged solution and the initial concentration (*C*_0_) of the above solution were monitored immediately using an ultraviolet-visible spectrometer. The absorption rate was calculated as *C*_t_/*C*_0_. The adsorption capacity (*q*_t_) was calculated by Equation (1).

(1) $$q_{t}\;=\;\frac{C_{0}-C_{t}}{m}V$$

## 3. Result and Discussion

### 3.1. The Structure and Adsorption Properties of CS/GO Aerogels Prepared by Three Experimental Methods

**Figure 2:**
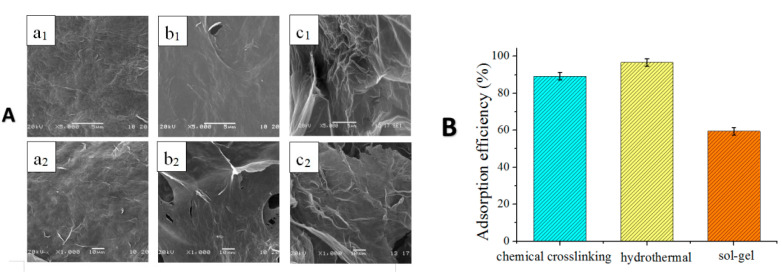
(**A**) SEM photographs of aerogels: (**a_1_**,**a_2_**) CS/GOc, (**b_1_**,**b_2_**) CS/GO_S_, and (**c_1_**,**c_2_**) CS/GO, (**B**) adsorption performance of CS/GO aerogels prepared by three experimental methods on methyl orange solution.

In this paper, CS/GO aerogels were prepared by hydrothermal, chemical cross-linking and sol-gel methods (a brief description is provided in the following sections), and were denoted as CS/GO, CS/GOc, and CS/GO_S_, respectively. Physical adsorption and desorption tests of N_2_, and SEM were used to characterize the structure of the three aerogels. Figure 2A shows the scanning electron microscopy of the three aerogels. The pore characteristics of the three aerogels are shown in Table 1. Obviously, it can be seen from the morphology that the CS/GO aerogel prepared by the hydrothermal method had more surface folds and more dense pores, which made the specific surface area of the CS/GO aerogel higher than CS/GOc and CS/GO_S_. Figure 2B shows the results of the MO-adsorption performances of CS/GO aerogels prepared by chemical crosslinking, hydrothermal, and sol-gel methods, respectively. The data demonstrate that the MO-adsorption efficiency of the CS/GO aerogel, prepared by the hydrothermal method, reached as far as 96.6%, and was significantly higher than that of the aerogels obtained by the chemical crosslinking method (89.2%) and sol-gel method (59.45%). This was because the high-pressure chemical environment of hydrothermal method [13] causes a series of benefits, including a three-dimensional structure, many holes, a large specific surface area, as well as superior adsorption for the synthesized CS/GO aerogel.

The study of CS/GO aerogels prepared by three experimental methods shows that the CS/GO aerogel prepared by the hydrothermal method was superior to the other two in terms of structure and adsorption performance. The following is a study of the properties of the CS/GO aerogel prepared by hydrothermal method and the control factors of the adsorption process.

### 3.2. FTIR Analyses of Various Aerogels

**Figure 3:**
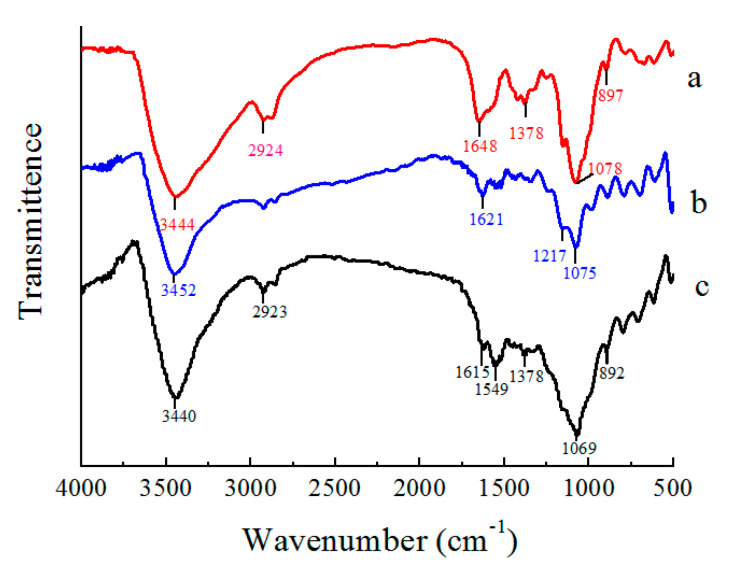
FT-IR spectra of aerogels: (**a**) CS, (**b**) GO, and (**c**) CS/GO.

To better understand the molecular structures of prepared aerogels of CS, GO, and CS/GO, the FTIR spectra was investigated and is displayed in Figure 3. For the CS aerogel, the peaks at around 1378 cm^−1^ and 897 cm^−1^ belong to the bending vibration of the C–H from –CH_3_ and the bean-ring structure of chitosan, respectively. Similarly, for the GO aerogel, except for the stretching vibration peak at nearly 3452 cm^−1^, the other peaks at 1621 cm^−1^ and 1075 cm^−1^ were, respectively, sourced from the factors of C=C and C–O in benzene. With regard to the CS/GO aerogel, it included not only the common characteristics from the bean-shaped appearance of chitosan at 892 cm^−1^ and C=C on graphene oxide at 1615 cm^−1^, but also the remaining strong bending vibration peak from –NH_2_ or –NH^3+^ at 1549 cm^−1^. This result indicates that the CS/GO aerogel can be fabricated through a chemical interaction between chitosan and graphene oxide.

### 3.3. XRD Analysis of Various Aerogels

**Figure 4:**
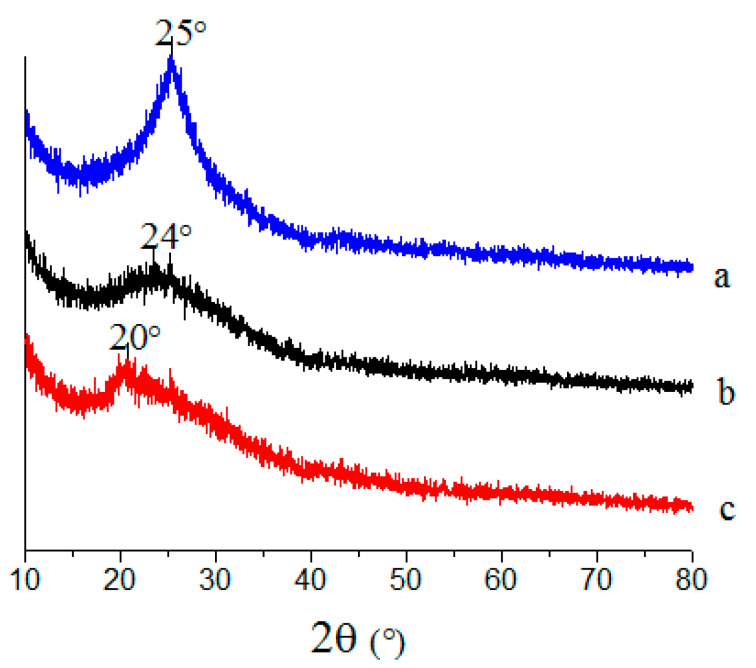
XRD patterns of aerogels: (**a**) GO, (**b**) CS/GO, and (**c**) CS.

The crystal structure of the CS, GO, and CS/GO aerogels was comparatively illustrated in corresponding XRD patterns, as shown in Figure 4. From the pattern of the CS aerogel, it is obvious that there is a diffraction peak emerging at 20°, which is attributed to the anhydrous crystal structure [14]. As for the GO aerogel, the diffraction peak appeared at 25°, and was associated with the interlayer spacing of 0.36 nm, calculated by the Bragg equation, whereas the peak intensity was not that strong. This results state that most of the oxygen-containing functional groups can be removed after hydrothermal reduction, and the resulting GO aerogels were randomly accumulated and distributed between graphene sheet layers. In addition, the diffraction peak at 20°, belonging to the structural characteristic of CS, disappeared and a new peak appeared at 24° in the pattern of the CS/GO aerogel, indicating that the addition of GO presents a great negative effect on the anhydrous crystal structure of chitosan. According to the Bragg equation, the interlayer spacing of the CS/GO aerogel was calculated as 0.37 nm, which shows less of an increase in contrast to the GO aerogel. This result indicates that the slight interlayer expansions of GO sheets happen due to the insertion of CS, leading to an effective preparation of CS/GO aerogel by way of a grafting modification.

### 3.4. SEM Analyses of Various Aerogels

**Figure 5:**
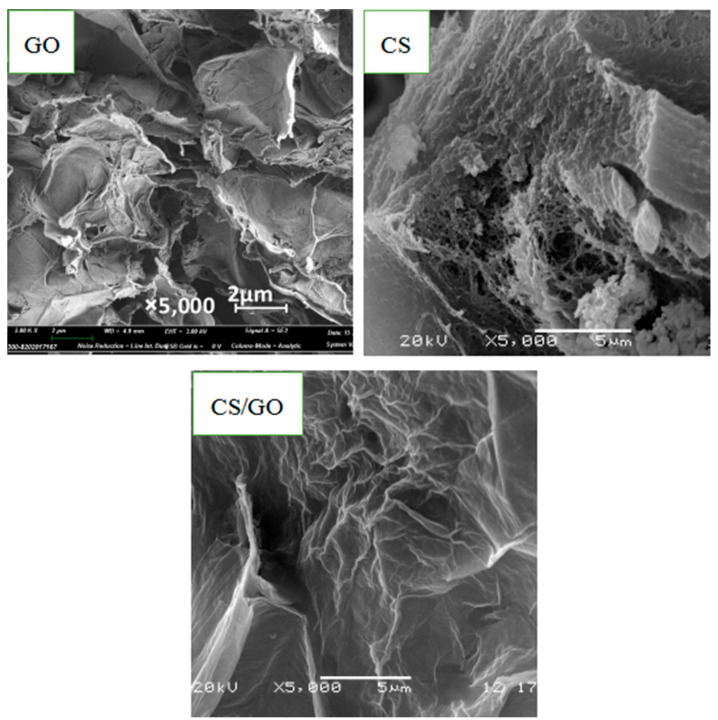
SEM photographs of GO aerogel, CS aerogel and CS/GO aerogel (5000×).

Figure 5 exhibits the SEM images of the CS aerogel, GO aerogel and CS/GO aerogel. As observed, the morphological characteristic of the GO aerogel is a three-dimensional structure, which reflects the typical structural nature of graphene’s lamellar folds where the space holes and smooth and compact surfaces simultaneously exist depending on the special structural effects of numerous folds. With respect to the surface morphology of the CS aerogel, it presents a relatively rough surface relating to amorphous dense accumulation, uneven voids, a loose porous structure, and fuzzy edges. For the CS/GO aerogel, the morphological appearance indicates that smaller aperture, narrower distribution, larger specific surface area, and denser holes were formed, which made the adsorption rate of the CS/GO aerogel higher than that of the CS aerogel and GO aerogel. In other words, the CS/GO aerogel prepared by this method had a uniform three-dimensional structure and was reliable for applications in the absorption of MO.

### 3.5. Physical Adsorption and Desorption Test of N_2_

**Figure 6:**
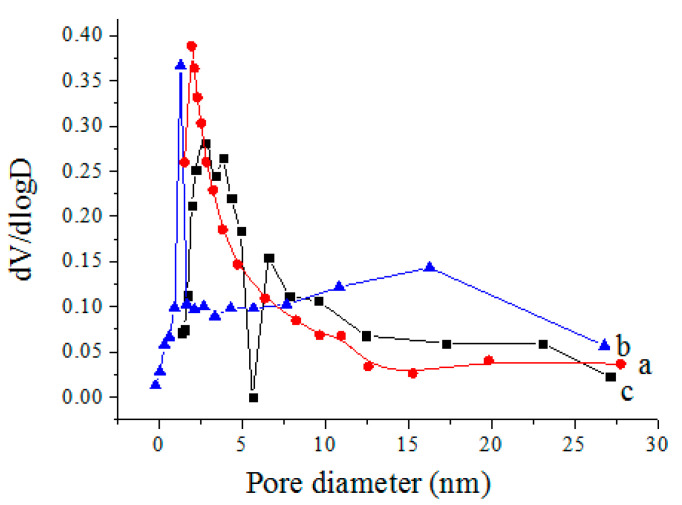
BJH pore size distribution curves of aerogels: (**a**) CS, (**b**) GO, and (**c**) CS/GO.

Figure 6 illustrates the BJH pore size distribution curves of the CS aerogel, GO aerogel and CS/GO aerogel, and the corresponding porous characteristics are presented in Table 2. It is clear that the BET value of the CS/GO aerogel reached 297.431 m^2^/g, which was significantly higher than that of the GO aerogel and CS aerogel. Meanwhile, the pore size of the CS/GO aerogel was the smallest among the studied aerogels. The reaction of CS and GO could not destroy the graphene skeleton structure. Chitosan was grafted on the surface of the graphene, reduced the reunion of graphene layers and formed a more dense hole, which was conducive to the free diffusion of dye molecules to increase opportunities for interaction with adsorption sites on the adsorbent [15]. Overall, the interaction between CS and GO contributed to the improvement of the specific surface area of the CS/GO aerogel in order to enhance its adsorption capacity.

### 3.6. Effect of Adsorbent Types

**Figure 7:**
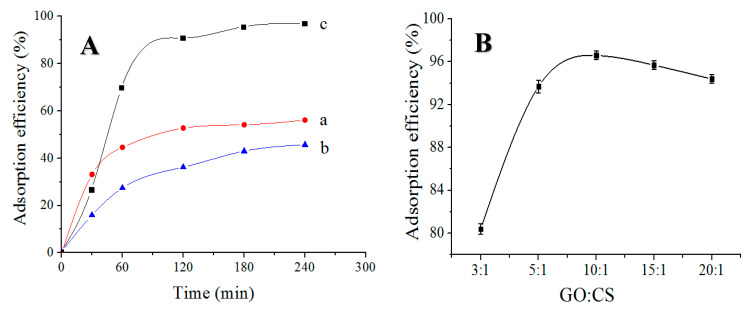
(**A**) Effect of adsorbent types on adsorption properties of methyl orange: (**a**) CS aerogel, (**b**) GO aerogel, (**c**) CS/GO aerogel, and (**B**) effect of the raw material ratio on the adsorption performance of the CS/GO aerogel.

50 mL solutions with initial concentrations (20 mg/L) were shaken for 5 h at room temperature with 20 mg of GO aerogel, CS aerogel CS/GO aerogel, respectively. Figure 7A shows that CS/GO aerogel had the best adsorption performance, with an adsorption rate of 95.3% and an adsorption capacity of 47.65 mg/g at 180 min. The oxygen-containing groups on the GO aerogel surface can absorb cationic dyes in water due to electrostatic action, so the adsorption rate of such anionic dyes as MO is not high—at only 45.3%. CS aerogel, due to its small specific surface area and limited adsorption sites on the aerogel, has a low adsorption rate (56.1%). Compared to pure GO aerogel, CS/GO aerogel prepared by the hydrothermal method improved the porosity. Due to the large amount of amino groups on the surface of the GS/GO aerogel, the adsorption point and specific surface area were increased, and the diffusion mechanism between the pollutants and adsorbents was improved, so the adsorption of the CS/GO aerogel was better than the other two unmodified adsorbents.

### 3.7. Effect of Raw Material Ratio on the Adsorption of CS/GO Aerogel

Figure 7B plots the MO-adsorption efficiency of the CS/GO aerogels with different mix proportions of GO and CS. In this study, various CS/GO aerogels were prepared with variables of mass ratio of GO to CS at 3:1, 5:1, 10:1, 15:1, and 20:1. For a brief description, as shown in the following sections, they are denoted as CS/GO_3_, CS/GO_5_, CS/GO_10_, CS/GO_15_, and CS/GO_20_, respectively. As the proportion of graphene oxide in the system increased, the adsorption rate increased. It is clear that the adsorption of CS/GO aerogel first increased and then decreased as the mass ratio of GO/CS rose. The results indicate that the adsorption efficiency of the CS/GO aerogel was largely dependent on the mass ratio of GO/CS, which could reach a peak value of nearly 96.6% when the mass ratio was fixed at around 10:1. This result reflects that the optimal porous microstructure and specific surface area of the synthesized CS/GO aerogel with the GO/CS mass ratio of 10:1 can be obtained to hold more MO molecules, in comparison to other mass ratios. If the mass ratio continued to be increased by more than 10:1, the adsorption response would present a slight decrease that was not expected for dye removals. The reason for this result may be that the excessive amounts of GO will cause the reduction of relative concentration of functional groups on the molecular structure of chitosan and thus negatively influence the absorption of the CS/GO aerogel.

### 3.8. Effect of pH

**Figure 8:**
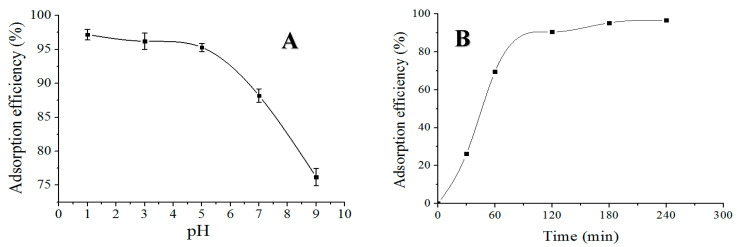
(**A**) Effect of pH value on adsorption performance of CS/GO aerogel. (**B**) Effect of adsorption time on adsorption performance of the CS/GO aerogel.

The pH value was also an important determinant affecting the adsorption of aerogels, which was designed to range from one to nine for the adsorption checking in this study [16]. The 50 mL methyl orange solution (20 mg/L) was used as the adsorption object, and the CS/GO_10_ aerogel (20 mg) was used as the adsorbent at room temperature. Figure 8A describes the MO-adsorption efficiency of the CS/GO aerogel with a changing pH value. It was found that the whole absorption of the CS/GO aerogel consistently decreased as the pH value increased. However, it is worthy to note that the adsorption efficiency remained relatively stable within the pH range of 1–5 and decreased rapidly when the pH value exceeded five. This depended upon the methyl orange having a kun-type structure in the acidic condition, and the sulfate end of its molecule being negatively charged, which could generate electrostatic adsorption with –NH^3+^ on chitosan to achieve the purpose of removing MO. Under alkaline conditions, methyl orange was negatively charged and was repulsive to the oxygen-containing functional groups of chitosan and graphene, causing the adsorption effect to behave less well than under acidic environments. In a word, the acidic chemical environments are beneficial to provide a high possibility for the prepared CS/GO aerogel to dispose and remove the dye in water, while its adsorption capacity can reach the maximum level with the adsorption efficiency of 97.2% and the adsorption capacity of 48.6 mg/g when the pH value is one.

### 3.9. Influence of Adsorption Time

Figure 8B presents the adsorption efficiency of the CS/GO aerogel with the elapsing time. It is significant that the adsorption rate of the CS/GO aerogel was fast in the early stage, within and around 120 min, and then increased slowly until reaching the adsorption equilibrium. The explanation for this is that the unique porous structure and advantageous that were specific to the surface area of the CS/GO aerogel provided a tremendous and special potential in the absorption of MO molecules, and the absorption will finish when the internal space of the CS/GO aerogel is totally filled.

### 3.10. Desorption

At present, the commonly used regeneration methods of adsorbent include the solvent method [17], heating method [18], electrochemical method [19], oxidation method [20], and more. In this experiment, the solvent method was adopted and 1.5 mol/L NaOH solution was used for desorption.

After the adsorption experiment, the remaining methyl orange solution was poured out. The adsorbent with distilled water was washed three times to remove the methyl orange that had not adsorbed firmly on the surface of the adsorbent. Then, the adsorbent was added to 30 mL of NaOH solution (1.5 mol/L) and placed in an oscillating water bath for 60 min. During this period, the color of the solution deepened, indicating that the dye molecules were gradually desorbing from the adsorbent. After full shock, the adsorbent was removed, washed with distilled water once, and then desorbed with NaOH solution one–two times until the solution became transparent, indicating that the dye on the adsorbent was basically removed. The remaining NaOH solution was removed from the desorbed adsorbent with distilled water (two–three times) until the solution was neutral. The desorbed adsorbent was dried to a constant weight at 60 °C for the next adsorption experiment.

**Figure 9:**
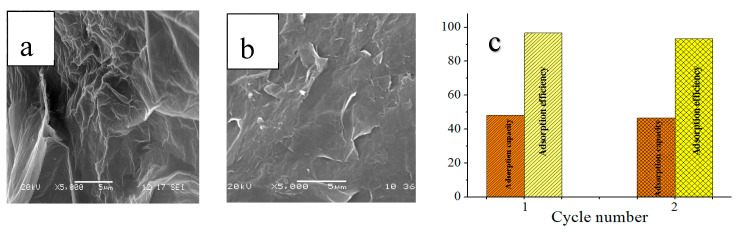
(**a**) SEM image of CS/GO aerogel before adsorption. (**b**) SEM image of CS/GO aerogel after desorption. (**c**) The effect of readsorption after desorption.

The structure of the CS/GO aerogel after desorption (Figure 9b) had no significant change compared to that before adsorption (Figure 9a). Figure 9c shows the adsorption effect of the second adsorption experiment after the first desorption. After desorption, the adsorption efficiency of the second adsorption was 93.24%, as compared to the first adsorption efficiency of 96.6%, and the adsorption effect was basically stable. This shows that the CS/GO aerogel can be recycled.

## 4. Adsorbing Mechanism Analysis of CS/GO Aerogel

### 4.1. Adsorption Kinetics Study

**Figure 10:**
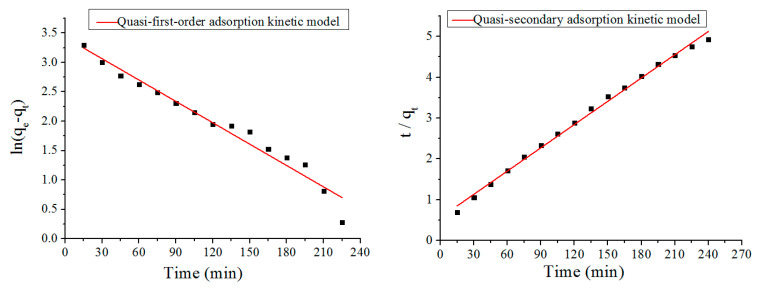
Fitting diagram of quasi-first-order adsorption kinetics model (**left**) and quasi-secondary adsorption kinetics model (**right**).

The adsorption data were fitted based on the quasi-first-order adsorption kinetics model (Equation (2)) and the quasi-second-order adsorption kinetics model (Equation (3)) [21], as shown in Figure 10, and the relevant parameters are presented in Table 3.

Quasi-first-order adsorption kinetic model:

(2) $$\ln(q_{e}-q_{t})=\ln q_{e}-k_{1}t$$

Quasi-secondary adsorption kinetic model:

(3) $$\frac{t}{q_{t}}=\frac{1}{k_{2}q_{e}{}^{2}}+\frac{t}{q_{e}}$$

In formula, *q*_e_ and *q*_t_ are adsorption equilibrium and adsorption capacity (mg/g) at time *t*, respectively; *t* is the adsorption time (min); and *k*_1_ and *k*_2_ represent the first-order adsorption kinetics constants (min^−1^) and the second-order adsorption kinetics constants (g·mg^−1^·min^−1^), respectively.

Table 3 shows the comparison of the regression coefficients, *R*^2^ (quasi-secondary) > *R*^2^ (quasi-first-order), and the maximum adsorption capacity calculated by the quasi-second-order adsorption kinetic model, which was 52.6 mg/g—close to the maximum adsorption capacity measured by the experiment of 48.68 mg/g. The result shows that the quasi-second-order adsorption kinetic model is more suitable to describe the adsorption process that is controlled by the active sites of chemisorption [22]. Meanwhile, the quasi-second-order kinetic model appears to be more reliable and real to reflect the adsorption capacity of the CS/GO aerogel on the MO molecules.

### 4.2. Isothermal Adsorption Model

Langmuir [23] and Freundlich [24] adsorption isotherm equations were used to evaluate the adsorption system and adsorption behavior.

The linear expression of Langmuir equation is as follows:

(4) $$\frac{C_{e}}{q_{e}}\;=\;\frac{C_{e}}{q_{\max}}+\frac{1}{k_{L}q_{\max}}$$

where *C*_e_—equilibrium mass concentration of the dye solution/(mg/L)

*q*_e_—equilibrium adsorption capacity/(mg/g)

*q*_max_—monolayer saturated adsorption capacity/(mg/g)

*k*_L_—Langmuir constant /L/mg related to adsorption capacity and adsorption rate

The basic characteristics of Langmuir adsorption isotherms can be represented by dimensionless equilibrium parameters (*R*_L_):

(5) $$R_{L}\;=\;\frac{1}{k_{L}C_{0}+1}$$

The Freundlich equation describes the adsorption process on an uneven surface. The equation is as follows:

(6) $$\ln\;q_{e}\;=\;\ln k_{F}+\frac{1}{n}\ln C_{e}$$

where *k*_F_ and *n* are Freundlich constants related to adsorption capacity and adsorption strength, respectively. The larger *k*_F_ and *n* values represent the adsorbent’s better adsorption performance. If (1/*n*) < 1, the adsorption conforms to the normal Langmuir isotherm model. If (1/*n*) > 1, collaborative adsorption occurs [25].

**Figure 11:**
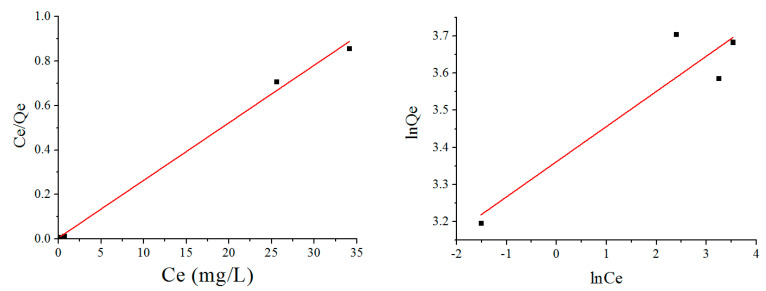
Langmuir adsorption isotherm (**left**) and Freundlich adsorption isotherm (**right**).

Langmuir and Freundlich adsorption isotherm equations were used to analyze the adsorption behavior of CS/GO aerogel to MO, as shown in Figure 11:

The data in Table 4 show that the correlation coefficient of the Langmuir equation (*R*^2^ = 0.9932) is higher than that of the Freundlich equation (*R*^2^ = 0.81199), so the former model is more suitable to reflect the adsorption. In addition, Table 5 presents the *R*_L_ value of equilibrium parameter at various concentrations, which can be used to judge the nature of the adsorption process [26]. The *R*_L_ values at various concentrations are all less than one, indicating that the adsorption of the CS/GO aerogels under the designed concentrations can easily happen. It is also worth noting that the R_L_ value decreased as the concentration rose, thus demonstrating that the chemical adsorption was more likely to occur at higher concentrations of the MO solution. Since the adsorption of MO is the electrostatic interaction between positively charged amino groups on the CS chain, the adsorption of dye molecules for the aerogel is the monolayer and the active sites are homogeneous. Moreover, according to the Freundlich equation, its constant of 1/*n* = 0.09478 was less than one, which indicates that the adsorption conforms to the normal Langmuir isotherm model.

### 4.3. Adsorption Mechanism

The adsorption mechanism of the graphene-like gels has been extensively studied. Zhang et al. [27] prepared GO/CS composites by chemical cross-linking, believing that materials with high porosity can provide enough space to absorb substances, facilitating material exchange. Singhi et al. [28] prepared chitosan-reduced graphene oxide(RGO) composite hydrogel for adsorption of organic dyes, suggesting that the initial dye concentration provides the necessary driving force to overcome the mass transfer resistance of the dyes at the active site of the adsorbent. Ruomeng, Yu et al. [29] prepared multifunctional GO/CS aerogel microspheres, which have good adsorption properties for heavy metal ions and dyes. It is believed that the -NH^3+^ group on the composite gel becomes the active site and is easily bound to the adsorption object through electrostatic interaction [30]. Yang et al. [31] constructed a novel 3D RGO aerogel. It is considered that the π–π and electrostatic interactions between aerogel and dye molecules and the synergistic effect of adsorbent polyhedron interface are the main driving factors of the ultra-high adsorption performance.

Both the GO aerogel and CS aerogel have the potential to be good adsorbents, but it is difficult to obtain satisfactory adsorption effects for the GO aerogel obtained by high temperature hydrothermal reaction, due to the mutual attraction and aggregation of the lamellar from the π–π action, and chitosan is easy to dissolve under acidic conditions, so it is not a practical adsorbent. Aromatic organic dyes, such as MO, tend to react with π–π action, so the structures, such as non-oxidized zone and aromatic, clustering on the GO, have a high affinity for them. Through the modification of GO surface graft CS, the three-dimensional network structure of graphene remains relatively intact, the increase of layer spacing between the GO lamellar layers, due to the insertion of CS, except that the lamellar spacing increases and the volume of holes in the middle increases, so that it can accommodate more pollutants. However, MO discharges negatively charged D-SO^3−^ in the aqueous phase. Due to the presence of abundant cationic groups on the surface of chitosan, it is easy to attract MO through electrostatic action, thus enhancing its adsorption performance.

The CS/GO aerogel is a three-dimensional porous structure that can be prepared through a simple and controllable hydrothermal method. Compared to other literatures (Table 6), it shows a larger specific surface area and a better adsorption effect.

**Figure 12:**
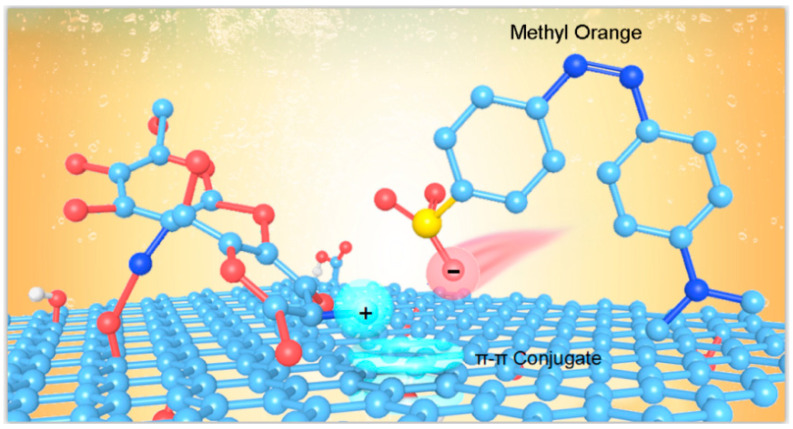
Schematic diagram of the adsorption of methyl orange by the CS/GO aerogel.

As mentioned above, the prepared CS/GO aerogel showed a good adsorption performance on MO. In Figure 12, compound gel on the GO has not been oxidized in the conjugate area shown in the π–π conjugate active adsorption sites, CS chain containing amino groups, with the H^+^ form –NH^3+^ in the water and positively charged, as positive active adsorption sites. MO are anionic dyed and the surface is negatively charged, based on the CS/GO composite aerogel on the adsorption of CS molecular chains of amino and dye molecules that electrostatically interact. Further, the conjugate effect between the unoxidized conjugate region of the GO and the dye molecules may also contribute to the increase in adsorption capacity. Therefore, the combination of electrostatic action and π–π conjugate action is the adsorption mechanism of the CS/GO composite aerogel.

## 5. Conclusions

This study mainly aimed to prepare a high-absorption CS/GO aerogel for application in removing methyl orange. Based on this, CS, GO, and CS/GO aerogels were, respectively, prepared through various methods in order to obtain comparative investigations of their microstructures and adsorption properties. Finally, some valuable and interesting findings were reached, as below:

(1) The hydrothermal method is an acceptable and applicable method to prepare the CS/GO aerogel with a high adsorption rate (96.6%), which proved to be better than the other approaches, such as the sol-gel method (59.45%) and chemical crosslinking method (89.2%);
(2) The microstructural characterizations indicate that the CS/GO aerogel has a combined structure provided by CS and GO aerogels, which has a larger specific surface area (297.431 m^2^/g), more micro-holes, and higher absorption volume;
(3) The experimental data of the CS/GO aerogel were well fitted by the Langmuir isotherm model in the case of MO. The quasi-secondary adsorption kinetic model provided a better correlation of the adsorption data than the quasi-first adsorption kinetic model, implying that the adsorption process is controlled by the active site of chemisorption;
(4) The CS/GO aerogel can enhance the adsorption capacity of MO and it can be well used for the adsorption of MO in a larger pH range (pH = 1~9); and
(5) CSGO is a promising adsorbent for the adsorption of MO in practice. Despite all this, this study mainly focused on the adsorption properties of CSGO for MO, and not for other sources of contaminants. Therefore, future research should be more concerned with cationic dyes and heavy metals.

## Figures and Tables

**Table 1:** Hole structure analysis of the aerogels.

Sample	BET (m^2^/g)	Pore Volume (cm^3^/g)	Pore Diameter (nm)
CS/GO_S_	56.916	0.267	15.275
CS/GO_c_	147.49	0.680	13.609
CS/GO	297.431	0.238	3.057

**Table 2:** Hole structure analysis of the samples.

Sample	BET (m^2^/g)	Pore Volume (cm^3^/g)	Pore Diameter (nm)
CS	88.993	0.1254	5.64
GO	31.88	0.137	8.287
CS/GO	297.431	0.238	3.057

**Table 3:** Results of CS/GO aerogel adsorption kinetics model fitting.

Model	*k*/(mg·g^−1^·min^−1^)	*q*_e_/(mg·g^−1^)	*R* ^2^
quasi-first-order adsorption kinetics model	0.01219	30.79	0.96239
quasi-secondary adsorption kinetics model	0.000641	52.6	0.99527

**Table 4:** Isothermal adsorption parameters of Langmuir and Freundlich.

Model	Langmuir			Freundlich		
*t*/°C	*q*_max_ (mg·g^−1^)	*k*_L_ (L/mg)	*R* ^2^	*k*_F_ (mg/g (L/mg)^1/*n*^)	*n*	*R* ^2^
25	38.67	5.002	0.9932	28.84	10.55	0.81199

**Table 5:** R_L_ value of methyl orange adsorbed by CS/GO aerogel.

Concentration (mg/L)	10	20	40	50
*R*_L_	0.0196	0.0099	0.00498	0.00398

**Table 6:** Comparison of adsorption of different adsorbents for MO [32,33,34,35,36,37,38].

Adsorbents	*q*_e_ (mg/g)	BET (m^2^/g)	Pore Diameter (nm)	Experimental Methods	References
GP55	30	453.4	3–4	Chemical crosslinking	[32]
RGO-MMT	32.5	43.17	123.28	Sol-gel method	[33]
M-CS/γ-Fe_2_O_3_/MWCNTs	31.44	—	—	Chemical crosslinking	[34]
CSGO	32.73	—	—	Sol-gel method	[35]
L-Cys-GA	75	154	3.7	Template method	[36]
GCAs	20	68.11	—	Chemical crosslinking	[37]
γ-Fe_2_O_3_/chitosan composite films	29.41	—	—	solution casting method	[38]
CS/GO	48.6	297.431	3	Hydrothermal method	this study
